# Questionnaire Study to Investigate the Preferences of Children, Parents, and Healthcare Professionals for Different Formulations of Oral Medicinal Products

**DOI:** 10.3390/pharmaceutics16040515

**Published:** 2024-04-08

**Authors:** Manfred Wargenau, Felicitas Baase, Kristin Eckardt, Lucas-Sebastian Spitzhorn, Sibylle Reidemeister, Ingrid Klingmann, Viviane Klingmann

**Affiliations:** 1 M.A.R.C.O. GmbH & Co. KG, Institute for Clinical Research and Statistics, Schirmerstrasse 71, 40211 Duesseldorf, Germany; manfred.wargenau@marco-institut.de (M.W.); kristin.eckardt@marco-institut.de (K.E.); lucas.spitzhorn@marco-institut.de (L.-S.S.); 2 Department of General Pediatrics, Neonatology and Pediatric Cardiology, Medical Faculty and University Hospital Düsseldorf, Heinrich Heine University Duesseldorf, 40225 Duesseldorf, Germany; febaa100@hhu.de; 3 Novartis Pharma AG, Global Drug Development/Technical Research & Development, Novartis Campus, 4056 Basel, Switzerland; sibylle.reidemeister@novartis.com; 4 Pharmaplex bv, Avenue Saint-Hubert 51, 1970 Wezembeek-Oppem, Belgium; iklingmann@pharmaplex.be

**Keywords:** mini-tablets, pediatric formulation, medicine dosage form, medicinal product administration

## Abstract

Since the acceptability of a medicine can significantly impact therapeutic outcomes, this study aimed to determine and compare the preferences of children, parents, and healthcare professionals for the most commonly used pediatric oral medicine formulations (syrup, mini-tablets, oblong tablets, round tablets) addressing all pediatric age groups, 0–<18 years (y). This survey study employed sex-, age-, and participant group-adapted questionnaires for eight cohorts of participants, i.e., children 6–<12 y, adolescents 12–<18 y, parents of children in four age groups (0–<2 y, 2–<6 y, 6–<12 y, and 12–<18 y), nurses, and pediatricians. Descriptive statistics were used for data analysis. In the age groups 0–<2 y and 2–<6 y, mini-tablets were preferred over syrup by all participants. In the age group 6–12 y, solid dosage forms were also preferred over syrup by all participants. In the age group 12–<18 y, healthcare professionals preferred solid dosage forms over syrup. Parents preferred higher amounts of mini-tablets and syrup compared to round and oblong tablets, while adolescents’ preferences did not differentiate between these formulations. Based on the study results and in contrast to current practice, it is suggested to consider solid dosage forms for future age-appropriate medicinal products already for younger age groups.

## 1. Introduction

Introducing new medicinal products for human use requires extensive studies ensuring their safety, quality, and efficacy in the target population. Especially for children (who are considered a vulnerable population), it is difficult to obtain relevant data. This possibly results in the administration of potentially inadequate medicinal products [1], increases the risks of insufficient treatment or adverse reactions, and deprives children of the full benefit of therapeutic advances.

Drug delivery has been described as a major question in the pediatric setting. Children are a very complex group due to the huge differences in the age range from newborns to adolescents. Appropriate physical and chemical formulations for these subgroups are needed [2]. Since such appropriate drug formulations are often missing, caregivers autonomously modify the tablets or use co-administration techniques, including mixing the medication with food to cover unpleasant tastes [3], thereby potentially influencing the therapeutic effect. In clinical practice, the specific pediatric requirements for adequate dosing depend on the age and physical development stage of the child. However, the major deficiencies involve the availability of licensed drug formulations in the required dose, the child’s ability to ingest standard-size solid dosage formulations, and the taste of oral medicines. This often results in the selection of an alternative formulation, e.g., liquid or suppository. Despite the importance of appropriate formulations in pharmacotherapy for children, there is limited factual knowledge about the use of dosage forms in current practice [4].

Thus, it is not only necessary to investigate the efficacy and optimal doses of pharmaceutical substances for different pediatric age groups but also to develop adapted galenic formulations to allow the most suitable routes of administration. Therefore, it is of high importance to identify formulations that are best accepted by the target group [5].

The patient’s perspective on a therapeutic approach, including the pharmaceutical medicinal product, has emerged as an important factor in achieving the desired effectiveness during pharmacotherapy. There is increasing evidence that the acceptability of a medicinal product and its use might have a significant impact on patients’ adherence to therapy, the perceived quality of life as well as the safety and benefit–risk profile of the medicine [6]. According to the European Medicines Agency’s definition, patient acceptability is “The overall ability and willingness of the patient to use and its care giver to administer the medicine as intended” [4] (p. 24). To ensure that a new pharmaceutical product is sufficiently acceptable for the use by patients and/or caregivers, relevant evidence for its suitability has to be generated during medicinal product development and reported in the submission dossier to the regulatory authorities. Since valid methods to evaluate acceptability are still fragmented and an internationally harmonized method has not yet been established, the choice of the methodology to evaluate acceptability is left to the applicant [4].

A number of studies have been performed investigating the acceptability of various formulations by more or less objective, validated-methodology-based investigations of swallowability and/or palatability in small children [7,8,9,10,11,12,13,14,15,16,17,18]. Interestingly, the current practice of liquid or syrup administration in children is considered to be unreliable, with significant under- or over-dosing [19]. Suitable solid oral dosage forms have been reported to have huge advantages, such as precise dosing and avoiding the problems associated with liquid formulations like drug stability, potentially toxic excipients, storage conditions [20], and taste-masking [17]. A variety of alternative pediatric formulations has been developed, such as mini-tablets with or without coating, in orodispersible or non-orodispersible form, oblong tablets, orodispersible films [21], oral suspensions, and powders [22].

For example, in previous studies of this working group, the suitability of the uncoated mini-tablets was demonstrated in various young age groups, including newborns, and also showed superiority compared to syrup [9,10,11,12]. Other studies have shown the acceptability of mini-tablets in children as well [17,23], which can also be administered in large numbers to achieve higher doses [11]. Also, very good acceptability and swallowability were shown for bigger tablets (oblong tablets, 2.5 × 6 mm) [12]. Even without experience in taking tablets, children aged 4–12 years (y) were able to swallow tablets sized from 6 to 10 mm without choking problems [24]. In light of this, it is of great importance that the revised version of the European Medicines Agency (EMA) Guideline from 2014 [4] no longer gives any age recommendations for solid oral dosage forms.

However, to promote patients’ perspectives in medicine development, the preferences expressed by relevant stakeholders are also an important aspect. The current knowledge about the preferences of older children and adolescents aged 6 to 18 y, parents, nurses, and pediatricians for different medicinal formulations is still limited. So far, only surveys assessing preferences for oral formulations in limited populations have been performed [25,26], and routine measures and criteria for acceptance in the pediatric setting are missing [27]. Mostly, simple scale approaches (visual analog scales, hedonic scales) have been employed. Additionally, Likert scales, preference or forced choice questionnaires/surveys, and observational methods of multiple parameters during the swallowing process have been used. Among the studies performed so far, palatability has been the most commonly investigated factor [28].

In this study, a questionnaire approach was selected since questionnaires are suitable, time-efficient, and precise methods to evaluate preferences [29]. To appropriately address pediatric subjects, complexity should be reduced, and selectable choices should be adapted to their specific cognitive abilities and language [30,31,32]. Following this strategy, children can better be included as active participants, giving first-hand insights for their own age group [31]. Since it is known that visualization of a Likert scale is preferred amongst children [29,33], we have employed this technique in the present study and adapted it for the reference group by including suitable smileys.

This questionnaire study investigated differences in preference after mere visual examination of various oral formulations by children, parents, nurses, and pediatricians for the most commonly used pediatric formulations for oral medicines, i.e., syrup, mini-tablets, and oblong and round tablets addressing all pediatric age groups from newborn to <18 y. To our knowledge, this is the first study with such a patient- and user-centric approach, targeting children in all age groups, parents of children in all age groups, and healthcare professionals involved in pediatric patient care under scientifically sound conditions.

## 2. Materials and Methods

### 2.1. Study Objectives

The primary objectives of this study were to identify the oral formulations most preferred by children (6–<12 y) and adolescents (12–<18 y) as well as the oral formulation most preferred by parents of children (0–<2 y and 2–<6 y). The secondary objectives were to identify the oral formulation most preferred by parents of children (6–<12 y) and adolescents (12–<18 y), and to identify the oral formulation considered best for different age groups by nurses and pediatricians. The endpoints addressing these objectives were the preferences of formulations assessed by (1) pairwise comparisons of formulations, (2) sorting formulations according to participants’ preference, (3) the children’s perceptions of taking the different kinds of formulations 3 times daily for 1 week, and (4) the parents’, nurses’, and pediatricians’ perceptions of giving the different kinds of formulations 3 times daily for 1 week to children and adolescents.

### 2.2. Study Design

For this questionnaire study, sex-, age- and participant-group-adapted questionnaires were used for a total of 8 cohorts with 30 participants per cohort. The cohorts consisted of children (6–<12 y), adolescents (12–<18 y), parents of children (0–<2 y, 2–<6 y, 6–<12 y, and 12–<18 y), nurses, and pediatricians. The participants had to answer all the questions in the respective questionnaire.

This study received a favorable opinion from the Ethics Committee of the Medical Faculty of the Heinrich Heine University Düsseldorf (No. 2022-1808, 13 January 2022), was registered in the German Clinical Trial Register (No. DRKS00027640), and was conducted according to the International Council for Harmonisation of Technical Requirements for Pharmaceuticals for Human Use’s standard of “Good Clinical Practice”.

All participants were recruited in January 2022. The participants were pediatric patients and parents of pediatric patients in the Department of General Pediatrics, Neonatology, and Pediatric Cardiology of the University Hospital Düsseldorf, Germany, as well as nurses and pediatricians who were employees of this department. Among pediatric patients aged 6–<18 y, 50% reported experience with regular medicine intake, of which about 80% reported that the medicine was an oral drug. In detail, 9 of 30 children aged 6–<12 y and 21 of 30 adolescents aged 12–<18 y had experience with regular intake of oral medicine. Also, more than 80% of parents of pediatric patients aged 6–<18 y reported regular administration of medicines to their child. Of these parents, about 80% reported experiences with oral medicines. Specifically, 9 of 30 parents had children aged 6–<12 y, and 11 of 30 parents had adolescents aged 12–<18 y who had experience with regular administration of oral medicines.

### 2.3. Inclusion/Exclusion Criteria

The following inclusion criteria applied: (1) pediatric patients aged 6–<18 y, of whom 50% had to be male, and parents of pediatric patients aged 0–<18 y who were approached independently of the pediatric patient’s health status, (2) pediatric patients aged 6–<18 y had to be able to read and understand the questionnaires, and (3) participants had to be capable of understanding the survey procedures and indicate their consent with a cross on the questionnaire. The exclusion criteria were as follows: (1) pediatric patients aged 6–<18 y unable to read and understand the questionnaire on their own (e.g., due to cerebral palsy), (2) pediatric patients in the postoperative period who were not yet fully awake, (3) pediatric patients that were not fully oriented regardless of the reason, and (4) participants not willing to complete the questionnaire.

### 2.4. Study Activities, Study Groups, and Formulations

After giving their consent, participants were assigned a unique identification number in chronological order of enrolment. All information and instructions were given in a standardized manner by the investigator using age-appropriate language. The participants did not ingest the various formulations but rated them only on a visual basis. Pediatric participants aged 6–<18 y were shown the formulations for their respective age group, i.e., mini-tablets (2 mm), syrup, and round and oblong tablets (Table 1). Parents were shown the formulations for the respective age group of their child. Nurses and pediatricians were shown all formulations. The participants completed the questionnaire in the presence of the investigator who showed them the different formulations. Questions for further clarification were possible at any time.

### 2.5. Sample Size and Statistics

The inclusion of 30 pediatric patients per age group and 30 parents of pediatric patients per children’s age group as well as 30 nurses and 30 pediatricians was considered appropriate for descriptive purposes. In total, 240 questionnaires (30 per cohort) were completed by participants and included in the analyses using descriptive statistical methods. The results are presented as frequency tables (including counts and percentages) for all evaluation criteria by cohort. Additionally, the differences between preference assessments by parents, nurses, and pediatricians were investigated.

### 2.6. Questionnaire Design

Four types of questionnaires were used to evaluate the preferences for the different formulations. Table 2 shows an overview of the questionnaire details.

**Figure 1:**
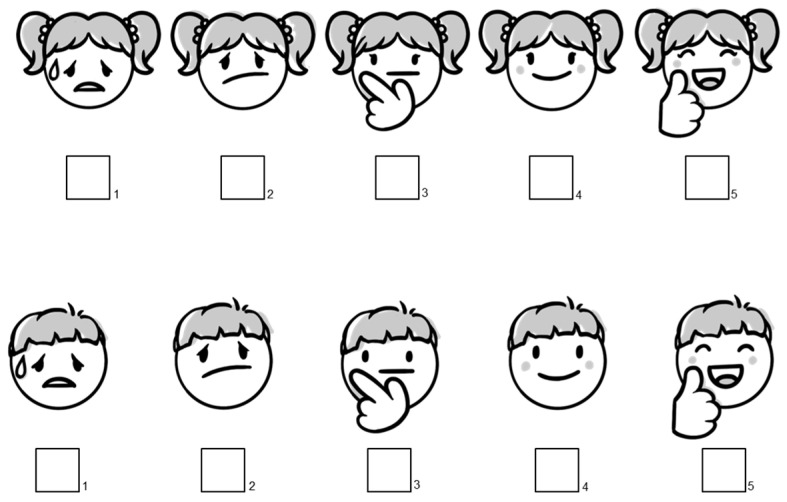
Sex-specific 5-point scales used for rating the 4 age-adapted formulation regimens in the questionnaires.

Figure 1 displays the sex-adapted 5-point scales used to rate the 4 age-adapted formulation regimens in the questionnaires.

## 3. Results

### 3.1. Age Group 0–<2 y

**Figure 2:**
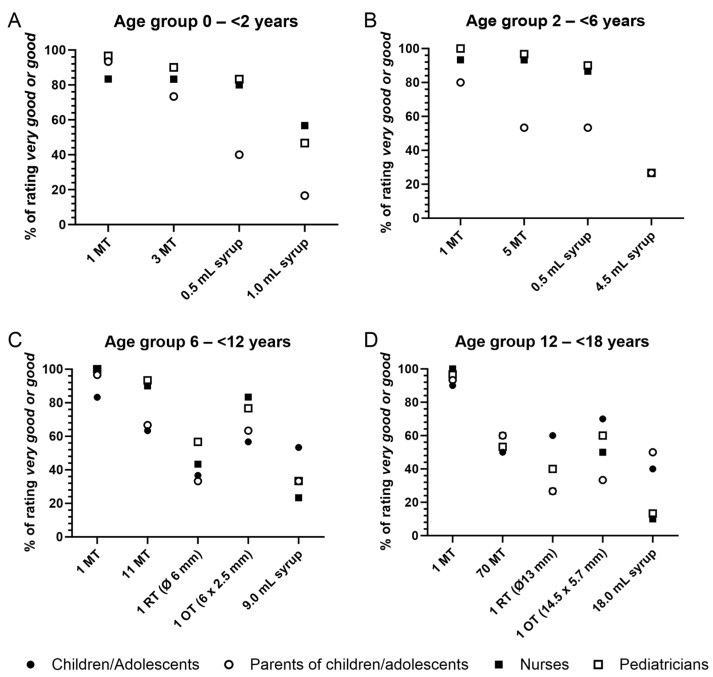
Ratings of formulations by age group and participant group. The graphs show the percentage of each participant group who rated the respective formulation as very good or good. (**A**) Age group 0–<2 y, (**B**) Age group 2–<6 y, (**C**) Age group 6–<12 y, (**D**) Age group 12–<18 y. MT = mini-tablet; OT = oblong tablet; RT = round tablet.

As shown in Figure 2A, 93% of parents rated one mini-tablet as very good or good, while three mini-tablets were considered as very good or good by 73% of the parents. The preference of parents was much lower for syrup, with 40% of parents rating 0.5 mL syrup and only 17% rating 1.0 mL syrup as very good or good (Table 3). Healthcare professionals assessed all formulations very similarly for the youngest age group. Mini-tablets were judged as very good or good by more than 80% of nurses and pediatricians, similar to 0.5 mL of syrup. The higher volume of syrup, i.e., 1 mL, was clearly rated as less favorable by both nurses and pediatricians. The assessments of both amounts of syrup by parents were clearly worse than those of nurses and pediatricians.

When comparing mini-tablets to syrup in a pairwise manner, a clear majority of parents preferred mini-tablets, especially when comparing three mini-tablets to 1.0 mL syrup (Table 4). Mini-tablets were chosen over syrup by a lower percentage of nurses and pediatricians compared to parents.

### 3.2. Age Group 2–<6 y

A total of 80% of parents judged one mini-tablet as very good or good (Figure 2B). Five mini-tablets were rated as very good or good by about half of the parents, similar to 0.5 mL syrup. However, only 27% of parents considered 4.5 mL syrup as very good or good (Table 3).

As for the youngest age group, healthcare professionals assessed all formulations very similarly. Both numbers of mini-tablets were rated as very good or good by >90% of healthcare professionals. The syrup volume of 0.5 mL was also judged as very good or good by the majority of nurses (87%) and pediatricians (90%), whereas the higher volume of 4.5 mL was considered less favorable.

When mini-tablets and syrup were compared pairwise, 70% of the parents, 80% to 90% of the nurses, and 90% of the pediatricians chose mini-tablets over syrup (Table 4).

### 3.3. Age Group 6–<12 y

In this age group, the rating of mini-tablets by children and parents was comparable (Figure 2C). One mini-tablet was considered as very good or good by 83% of children and 97% of parents. About 65% of children and parents judged the higher number of mini-tablets as very good or good. Also, for the round and oblong tablets, the rating results were similar for children and parents. Syrup was considered very good or good by 53% of children but only by 33% of parents. Again, nurses and pediatricians rated the formulations similarly. Mini-tablets were assessed as very good or good by 100% (1 mini-tablet) and more than 90% (11 mini-tablets) of healthcare professionals. The oblong tablet was rated as very good or good by about 80% of nurses and pediatricians, whereas syrup was judged as very good or good by only 23% and 33% (Table 3).

In the pairwise comparison of formulations, mini-tablets were favored over round tablets by comparable percentages of children, parents, and healthcare professionals (53% to 73%, Table 4). Mini-tablets were also favored over syrup by 70% to 77% of children, parents, and nurses and by 93% of pediatricians. When comparing mini-tablets to the oblong tablet, about half of the children and parents chose mini-tablets, while about 70% of healthcare professionals favored the oblong tablet over mini-tablets. Interestingly, the oblong tablet was also favored over the round tablet and syrup by more than 60% of all participant groups, especially by healthcare professionals.

**Figure 3:**
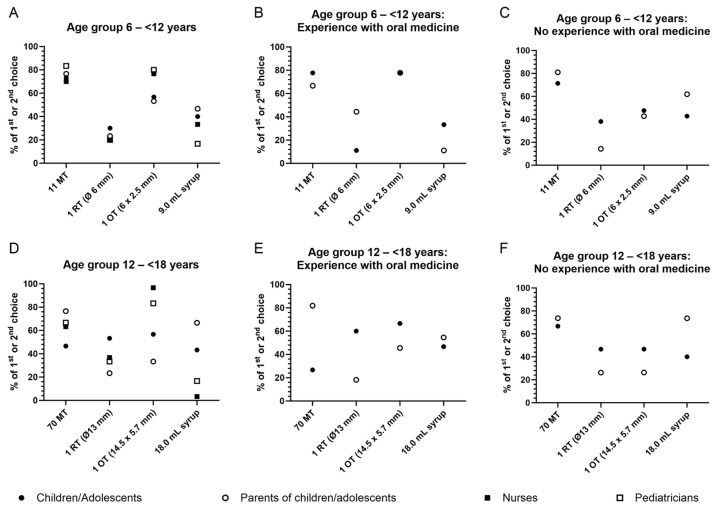
Ranking of formulations by age group and participant group. The graphs show the percentage of each participant group who ranked the respective formulation as 1st or 2nd choice (**A**) Age group 6–<12 y, (**D**) Age group 12–<18 y, as well as the percentage of children/adolescents and parents of children/adolescents with (**B**) Age group 6–<12 y, (**E**) Age group 12–<18 y or without experience of regular oral medicine intake (**C**) Age group 6–<12 y, (**F**) Age group 12–<18 y). MT = mini-tablet; OT = oblong tablet; RT = round tablet.

**Figure 4:**
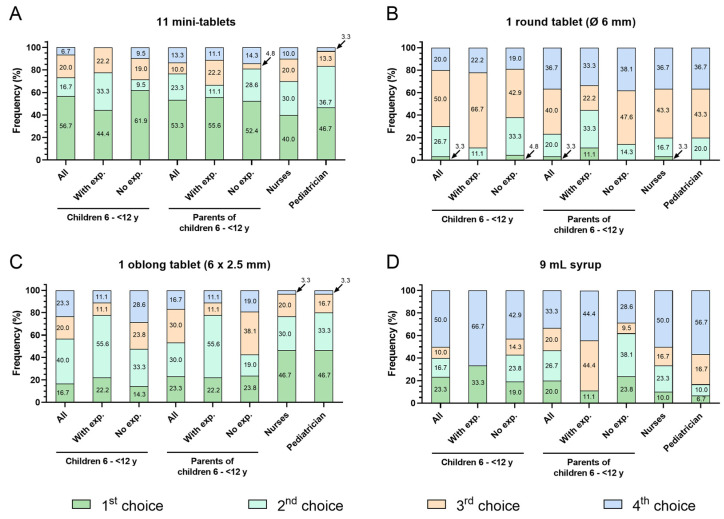
Ranking of formulations in the age group 6–<12 y by participant groups. The graphs show the percentage of each participant group who ranked the respective formulation (**A**) 11 mini-tablets, (**B**) 1 round tablet (Ø 6 mm), (**C**) 1 oblong tablet (6 × 2.5 mm), (**D**) 9 mL syrup as 1st, 2nd, 3rd, or 4th choice. For children and parents of children, the percentage is given for all participants of these 2 groups and separately by experience with regular oral medicine intake. exp. = experience.

Finally, participants were asked to rank all formulations according to their preference. Figure 3A shows the percentage of participants per group who indicated the respective formulation as their first or second choice. The ranking of mini-tablets and round tablets was very similar between all participant groups. Between 70% and 83% of children, parents, and healthcare professionals selected mini-tablets, while between 20% and 30% chose the round tablet as their first or second choice. The oblong tablet was named the first or second choice by about 50% of children and parents and by about 80% of nurses and pediatricians. Syrup was ranked as first or second choice by 40% of the children, 47% of parents, and 33% of nurses but only by 17% of pediatricians (Figure 3A and Figure 4A–D).

Taking into consideration the experience of children with regular intake of oral medicines, the selection of mini-tablets and syrup as first or second choice was similar among those with or without experience (Figure 3B,C and Figure 4A,D). Only about 11% of children with experience of regular oral medicine intake named the round tablet as their first or second choice, but 38% of children without such experience did so. The oblong tablet was selected as the first or second choice by significantly more children with experience compared to inexperienced children (78% and 48%) (Figure 3B,C and Figure 4B,C).

The selection of mini-tablets as first or second choice by parents of children with or without experience of regular oral medicine administration was comparable (67% and 81%). More parents of experienced children selected the round and oblong tablet (44% and 78%) as compared to those of children with no experience (14% and 43%). Regarding syrup, only 11% of parents with children experienced in regular oral medicine intake ranked this formulation as their first or second choice but 43% of parents with inexperienced children did so (Figure 3B,C and Figure 4A–D).

### 3.4. Age Group 12–<18 y

In the oldest age group (Figure 2D), the assessment of mini-tablets was similar for all participant groups, with 90% to 100% of participants rating 1 mini-tablet and 50% to 60% rating 70 mini-tablets as very good or good (Table 3). For the other formulations, the differences in ratings were more pronounced. One round tablet was judged as very good or good by 60% of adolescents, 40% of pediatricians, and 27% of parents and nurses. Between 33% (parents) and 70% (adolescents) of participants considered one oblong tablet as very good or good. For syrup, 40% of adolescents and 50% of parents rated it as very good or good, but only about 10% of both healthcare professional groups.

When comparing the formulations pairwise, 43% of adolescents favored mini-tablets over the round tablet and the round tablet over the oblong tablet (Table 4). The oblong tablet was favored over mini-tablets and syrup by about 60% of the adolescents. In contrast, more than 70% of the parents favored mini-tablets over round and oblong tablets. Only 33% of parents chose oblong over round tablets. Nurses clearly chose the oblong tablet over mini-tablets (73%), the round tablet (97%), and syrup (93%), and favored mini-tablets (87%) and the round tablet (83%) over syrup. Also, pediatricians chose the oblong tablet over mini-tablets (60%), round tablets (87%), and syrup (80%).

**Figure 5:**
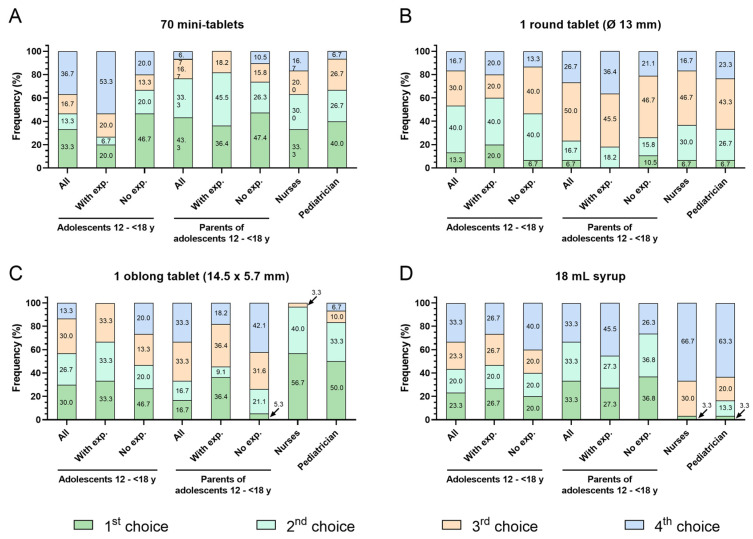
Ranking of formulations in the age group 12–<18 y by participant groups. The graphs show the percentage of each participant group who ranked the respective formulation (**A**) 70 mini-tablets, (**B**) 1 round tablet (Ø 13 mm), (**C**) 1 oblong tablet (14.5 × 5.7 mm), (**D**) 18 mL syrup as 1st, 2nd, 3rd, or 4th choice. For adolescents and parents of adolescents, the percentage is given for all participants of these 2 groups and separately by experience with regular oral medicine intake. exp. = experience.

Obvious differences in ranking the formulations were observed for adolescents compared to parents and health professionals, while nurses and pediatricians scored comparably (Figure 3D). A total of 77% of parents and about 65% of healthcare professionals named mini-tablets as their first or second choice, but only 47% of adolescents. The round tablet was selected as the first or second choice by 53% of adolescents, 23% of parents, and about 35% of healthcare professionals. The oblong tablet was named as first or second choice by 57% of adolescents, 33% of parents, and more than 80% of healthcare professionals. Syrup was selected as the first or second choice by 43% of adolescents and 67% of parents but only by 3% of nurses and 17% of pediatricians (Figure 3D and Figure 5A–D).

Taking into consideration the experience of adolescents with regular intake of oral medicines (Figure 3E,F), mini-tablets were selected as the first or second choice by only 27% of adolescents with experience but by 67% of adolescents with no experience. More adolescents with experience of regular oral medicine intake named the round and oblong tablet as their first or second choice (60% and 67%) compared to adolescents with no experience (47% for both the round and oblong tablets). The ranking of syrup as first or second choice was comparable between adolescents with or without experience (Figure 3E,F and Figure 5A–D).

The selection of mini-tablets as first or second choice was comparably high among parents of adolescents with or with no experience of regular oral medicine administration (82% and 74%). A similar observation was made for the round tablet, although significantly fewer parents of experienced and not experienced adolescents selected it as their first or second choice (18% and 26%). A total of 46% of parents with adolescents who had experience named the oblong tablet as their first or second choice, compared to 26% of parents with adolescents who had no experience. Syrup was named as the first or second choice by 55% of parents with experienced adolescents and by 74% of parents with adolescents who had no experience (Figure 3E,F and Figure 5A–D).

## 4. Discussion

In the age groups 0–<2 y and 2–<6 y, mini-tablets were preferred over syrup, and the higher amount of syrup was the least preferred formulation in these age groups rated by all stakeholders. The preferences expressed by nurses and pediatricians for the formulations in these two age groups were very consistent. Parents clearly preferred one mini-tablet, similar to the healthcare professionals. However, parents’ preference for a higher number of mini-tablets and syrup was lower than that of healthcare professionals. This difference might be related to the experiences of parents during regular applications in at-home and daily life settings.

Since it is known that children at the age of 6 y are able to control the swallowing process comparably to adults [34], in the age groups 6 < 12 y and 12–<18 y, commonly marketed standard oblong/round tablets were also investigated in addition to mini-tablets and syrup. In general, in the age group 6–<12 y, solid dosage forms were preferred over a liquid formulation by all stakeholders.

Within the group of solid dosage forms, mini-tablets and an oblong tablet were rated higher than a round tablet. The preferences of healthcare professionals were consistent, as were those of children and parents. Comparing these two groups, healthcare professionals rated the preference for an oblong tablet higher compared to children and parents and the preference for syrup lower than children and parents. Interestingly, only children had a comparable preference for oblong tablets and syrup, which might be related to their experience level. More importantly, children experienced in the regular intake of oral medicines clearly preferred the oblong tablet over all other formulations. Parents with children experienced in the regular intake of oral medicines had a very low preference for syrup.

In the age group 12–<18 y, healthcare professionals preferred solid dosage forms over syrup and rated oblong tablets comparably to the higher number of mini-tablets. Again, nurses’ and pediatricians’ preferences for the different formulations were consistent. Parents preferred higher amounts of mini-tablets and syrup compared to round and oblong tablets in this age group, while adolescents’ preferences did not differentiate between these formulations. Of note, the preference for mini-tablets of adolescents experienced in the regular intake of oral medicines was significantly lower compared to inexperienced adolescents.

The primary objectives of this study were to identify the most preferred oral formulation rated by children aged 6–<12 y and adolescents 12–<18 y as well as the most preferred oral formulation rated by parents of children 0–<2 y and 2–<6 y. For children, the most preferred formulations were higher amounts of mini-tablets and oblong tablets. For adolescents, there was no clear preference for any of the formulations, and thus, the most preferred oral formulation could not be identified. Parents of children 0–<2 y preferred mini-tablets over syrup, and parents of children 2–<6 y preferred mini-tablets (different amounts) and the lower quantity of syrup over the higher syrup volume. These results are in line with the preferences for future drug applications expressed by parents of children between the ages of 6 and 11 months in another study [23].

The secondary objectives were to identify the most preferred oral formulation rated by parents of children 6–<12 y and 12–<18 y, and to identify the oral formulation considered best for different age groups by nurses and pediatricians. Parents of children preferred mini-tablets and oblong tablets over round tablets and syrup. Parents of adolescents preferred higher amounts of mini-tablets and syrup over round and oblong tablets. Nurses and pediatricians considered mini-tablets (different amounts) and a low volume of syrup best for children 0–<2 y and 2–6 y. They considered mini-tablets and oblong tablets best suited for children 6–< 12 y, and oblong tablets for adolescents.

The study population comprised pediatric patients aged 0–<18 y, parents, nurses, and pediatricians of the Department of General Pediatrics, Neonatology, and Pediatric Cardiology of the University Hospital Düsseldorf, Germany. For the study, a total of 240 evaluable questionnaires (30 per cohort) were included. Though the population of Düsseldorf is mixed with respect to race, the study was conducted only at one site and in one country. To generalize the results, this kind of study should be repeated at more sites and in different countries/regions. Thus, maybe our methodology can contribute to the development of an internationally harmonized method for acceptability testing in children. A further improvement in understanding medicines’ acceptability would be not only beneficial for patients but also for the drug development process [28].

Overall, a general preference for solid dosage forms over liquid formulations (syrup) was observed across all age groups. This preference was consistent among all stakeholders, despite some differences in formulation preferences of different stakeholders for the four age groups of pediatric patients. This very important result is in line with several other investigations and studies [8,9,10,11,12,14,15,16,17,18,23] and contrasts with the current practice of administering medicines to children aged 0–<6 y as a liquid formulation. As syrups or other liquid formulations might contain excipients with potential toxicological risks [35], a switch to alternative dosage forms could not only increase the acceptability but also improve the risk profile.

Based on the results of this study, it is suggested to consider solid dosage forms for future age-appropriate medicinal products already for younger age groups, e.g., young children.

## Figures and Tables

**Table 1:** Overview of formulations.

Age Group	Formulations			
	No. of MT	Size of RT	Size of OT	Max. Vol. of Syrup
0–<2 y	1 and 3	n.a.	n.a.	1.0 mL
2–<6 y	1 and 5	n.a.	n.a.	4.5 mL
6–<12 y	1 and 11	Ø 6 mm	6 × 2.5 mm	9.0 mL
12–<18 y	1 and 70	Ø 13 mm	14.5 × 5.7 mm	18.0 mL
Manufacturer	NextPharma, Göttingen, Germany			Caesar & Loretz GmbH, Hilden, Germany
Ingredients	Lactose, cellulose, magnesium stearate, anhydrous colloidal silicon dioxide			Glucose, water

Max. = maximum; MT = mini-tablet(s), 2 mm; n.a. = not applicable; OT = oblong tablet; RT = round tablet; vol. = volume; y = years of age; Ø = diameter.

**Table 2:** Overview of questionnaires.

Type	Group	Questions
A	6–<18 y	(1) Previous experience with the formulations (“Ever taken?”: yes/no); (2) Preference for one formulation in a pairwise comparison with the other formulations (a total of 6 pairs possible); (3) Ranking of the 4 formulations according to their preference (from 1 = best to 4 = worst); (4) Rating of each formulation (“How much would you like to take this formulation 3 times daily for 1 week?’’) according to a 5-point scale presented as sex-specific smileys (Figure 1).
B	Parents of 0–<6 y	(1) Previous experience with the formulations (“Ever given to the child?”: yes/no); (2) Pairwise preference when comparing 1 mini-tablet vs. 0.5 mL syrup as well as the age-adapted maximal number of mini-tablets vs. the age-adapted maximal volume of syrup (Table 1); (3) Rating of the 4 age-adapted formulations regimens (“How much would you like to give this formulation to your child 3 times daily for 1 week?”) according to a 5-point scale presented as sex-specific smileys; The formulation regimens presented were 1 mini-tablet, 3 or 5 mini-tablets, 0.5 mL syrup, and 1 mL or 4.5 mL syrup.
C	Parents of 6–<18 y	(1) Previous experience with the formulations (“Ever given to the child/adolescent?”: yes/no); (2) Preference for one formulation in a pairwise comparison with the other formulations (a total of 6 pairs possible); (3) Ranking of the 4 formulations according to their preference (from 1 = best to 4 = worst); (4) Rating of each formulation (“How much would you like to take this formulation 3 times daily for 1 week?’’).
D	Nurses and Pediatricians	(1) Years of experience in the pediatric area categorized as 0–<5 y, 5–<10 y, and ≥10 y; (2) Previous experience (yes/no) with medicines given as mini-tablets, syrup, round tablet, or oblong tablet for each age group of pediatric patients; (3) Preference for one formulation in a pairwise comparison with the other formulations (a total of 6 pairs possible), specifically for each age group of pediatric patients; (4) Ranking of the 4 formulations according to their preference (from 1 = best to 4 = worst) for pediatric patients aged 6–<12 y and 12–<18 y; (5) Rating of each formulation (“How much would you like to give this formulation to children 3 times daily for 1 week?”) according to a 5-point scale presented as smileys, specifically for each age group of pediatric patients.

**Table 3:** Ratings of formulations by children’s age group and participant groups.

Participant Group	Formulation	Rating				
		Very Bad	Bad	Indifferent	Good	Very Good
Age group 0–<2 years						
Parents of children N = 30 (100%)	1 MT	–	–	2 (6.7%)	11 (36.7%)	17 (56.7%)
	3 MT	–	2 (6.7%)	6 (20.0%)	8 (26.7%)	14 (46.7%)
	0.5 mL syrup	–	6 (20.0%)	12 (40.0%)	7 (23.3%)	5 (16.7%)
	1.0 mL syrup	10 (33.3%)	9 (30.0%)	6 (20.0%)	4 (13.3%)	1 (3.3%)
Nurses N = 30 (100%)	1 MT	–	1 (3.3%)	4 (13.3%)	5 (16.7%)	20 (66.7%)
	3 MT	1 (3.3%)	1 (3.3%)	3 (10.0%)	7 (23.3%)	18 (60.0%)
	0.5 mL syrup	–	1 (3.3%)	5 (16.7%)	14 (46.7%)	10 (33.3%)
	1.0 mL syrup	–	3 (10.0%	10 (33.3%)	14 (46.7%)	3 (10.0%)
Pediatricians N = 30 (100%)	1 MT	1 (3.3%)	–	–	7 (23.3%)	22 (73.3%)
	3 MT	1 (3.3%)	–	2 (6.7%)	9 (30.0%)	18 (60.0%)
	0.5 mL syrup	–	1 (3.3%)	4 (13.3%)	13 (43.3%)	12 (40.0%)
	1.0 mL syrup	1 (3.3%)	1 (3.3%)	14 (46.7%)	9 (30.0%)	5 (16.7%)
Age group 2–<6 years						
Parents of children N = 30 (100%)	1 MT	–	1 (3.3%)	5 (16.7%)	16 (53.3%)	8 (26.7%)
	5 MT	–	7 (23.3%)	7 (23.3%)	11 (36.7%)	5 (16.7%)
	0.5 mL syrup	–	7 (23.3%)	7 (23.3%)	14 (46.7%)	2 (6.7%)
	4.5 mL syrup	6 (20.0%)	11 (36.7%)	5 (16.7%)	6 (20.0%)	2 (6.7%)
Nurses N = 30 (100%)	1 MT	–	1 (3.3%)	1 (3.3%)	5 (16.7%)	23 (76.7%)
	5 MT	1 (3.3%)	–	1 (3.3%)	9 (30.0%)	19 (63.3%)
	0.5 mL syrup	–	1 (3.3%)	3 (10.0%)	13 (43.3%)	13 (43.3%)
	4.5 mL syrup	1 (3.3%)	10 (33.3%)	11 (36.7%)	8 (26.7%)	–
Pediatricians N = 30 (100%)	1 MT	–	–	–	3 (10.0%)	27 (90.0%)
	5 MT	–	–	1 (3.3%)	9 (30.0%)	20 (66.7%)
	0.5 mL syrup	–	–	3 (10.0%)	14 (46.7%)	13 (43.3%)
	4.5 mL syrup	1 (3.3%)	6 (20.0%)	15 (50.0%)	6 (20.0%)	2 (6.7%)
Age group 6–<12 years						
Children N = 30 (100%)	1 MT	1 (3.3%)	–	4 (13.3%)	7 (23.3%)	18 (60.0%)
	11 MT	–	3 (10.0%)	8 (26.7%)	11 (36.7%)	8 (26.7%)
	1 RT ^a^	5 (16.7%)	4 (13.3%)	10 (33.3%)	6 (20.0%)	5 (16.7%)
	1 OT ^b^	1 (3.3%)	5 (16.7%)	7 (23.3%)	9 (30.0%)	8 (26.7%)
	9.0 mL syrup	3 (10.0%)	5 (16.7%)	6 (20.0%)	8 (26.7%)	8 (26.7%)
Parents of children N = 30 (100%)	1 MT	–	1 (3.3%)	–	10 (33.3%)	19 (63.3%)
	11 MT	–	4 (13.3%)	6 (20.0%)	11 (36.7%)	9 (30.0%)
	1 RT ^a^	3 (10.0%)	6 (20.0%)	11 (36.7%)	9 (30.0%)	1 (3.3%)
	1 OT ^b^	–	6 (20.0%)	5 (16.7%)	11 (36.7%)	8 (26.7%)
	9.0 mL syrup	5 (16.7%)	6 (20.0%)	9 (30.0%)	7 (23.3%)	3 (10.0%)
Nurses N = 30 (100%)	1 MT	–	–	–	4 (13.3%)	26 (86.7%)
	11 MT	–	–	3 (10.0%)	13 (43.3%)	14 (46.7%)
	1 RT ^a^	–	5 (16.7%)	12 (40.0%)	10 (33.3%)	2 (6.7%)
	1 OT ^b^	–	1 (3.3%)	4 (13.3%)	20 (66.7%)	5 (16.7%)
	9.0 mL syrup	1 (3.3%)	9 (30.0%)	13 (43.3%)	5 (16.7%)	2 (6.7%)
Pediatricians N = 30 (100%)	1 MT	–	–	–	2 (6.7%)	28 (93.3%)
	11 MT	–	–	2 (6.7%)	11 (36.7%)	17 (56.7%)
	1 RT ^a^	–	3 (10.0%)	10 (33.3%)	14 (46.7%)	3 (10.0%)
	1 OT ^b^	–	1 (3.3%)	6 (20.0%)	12 (40.0%)	11 (36.7%)
	9.0 mL syrup	–	3 (10.0%)	17 (56.7%)	8 (26.7%)	2 (6.7%)
Age group 12–<18 years						
Adolescents N=30 (100%)	1 MT	–	1 (3.3%)	2 (6.7%)	6 (20.0%)	21 (70.0%)
	70 MT	3 (10.0%)	1 (3.3%)	11 (36.7%)	11 (36.7%)	4 (13.3%)
	1 RT ^c^	1 (3.3%)	4 (13.3%)	7 (23.3%)	14 (46.7%)	4 (13.3%)
	1 OT ^d^	2 (6.7%)	2 (6.7%)	5 (16.7%)	15 (50.0%)	6 (20.0%)
	18.0 mL syrup	3 (10.0%)	7 (23.3%)	8 (26.7%)	8 (26.7%)	4 (13.3%)
Parents of adolescents N = 30 (100%)	1 MT	1 (3.3%)	–	1 (3.3%)	6 (20.0%)	22 (73.3%)
	70 MT	–	2 (6.7%)	10 (33.3%)	13 (43.3%)	5 (16.7%)
	1 RT ^c^	2 (6.7%)	12 (40.0%)	8 (26.7%)	6 (20.0%)	2 (6.7%)
	1 OT ^d^	2 (6.7%)	11 (36.7%)	6 (20.0%)	9 (30.0%)	2 (6.7%)
	18.0 mL syrup	4 (13.3%)	7 (23.3%)	4 (13.3%)	8 (26.7%)	7 (23.3%)
Nurses N = 30 (100%)	1 MT	–	–	–	4 (13.3%)	26 (86.7%)
	70 MT	1 (3.3%)	2 (6.7%)	9 (30.0%)	11 (36.7%)	7 (23.3%)
	1 RT ^c^	5 (16.7%)	8 (26.7%)	9 (30.0%)	5 (16.7%)	3 (10.0%)
	1 OT ^d^	–	4 (13.3%)	11 (36.7%)	11 (36.7%)	4 (13.3%)
	18.0 mL syrup	13 (43.3%)	8 (26.7%)	6 (20.0%)	2 (6.7%)	1 (3.3%)
Pediatricians N = 30 (100%)	1 MT	–	1 (3.3%)	–	2 (6.7%)	27 (90.0%)
	70 MT	1 (3.3%)	2 (6.7%)	11 (36.7%)	9 (30.0%)	7 (23.3%)
	1 RT ^c^	2 (6.7%)	7 (23.3%)	9 (30.0%)	9 (30.0%)	3 (10.0%)
	1 OT ^d^	1 (3.3%)	3 (10.0%)	8 (26.7%)	11 (36.7%)	7 (23.3%)
	18.0 mL syrup	5 (16.7%)	11 (36.7%)	10 (33.3%)	3 (10.0%)	1 (3.3%)

^a^: Ø 6 mm; ^b^: 6 × 2.5 mm; ^c^: Ø 13 mm; ^d^: 14.5 × 5.7 mm; N = number of participants; MT = mini-tablet(s); OT = oblong tablet; RT = round tablet; Ø = diameter.

**Table 4:** Pairwise preference comparison of formulations by children’s age group and participant groups.

Age Group (Years)	Preference Comparison	Children N = 30 (100%)	Parents of Children N = 30 (100%)	Nurses N = 30 (100%)	Pediatricians N = 30 (100%)
0–<2	1 MT vs. 0.5 mL syrup	–	23 (76.7%)	17 (56.7%)	20 (66.7%)
	3 MT vs. 1.0 mL syrup	–	28 (93.3%)	19 (63.3%)	20 (66.7%)
2–<6	1 MT vs. 0.5 mL syrup	–	21 (70.0%)	24 (80.0%)	27 (90.0%)
	5 MT vs. 5.0 mL syrup	–	21 (70.0%)	27 (90.0%)	27 (90.0%)
6–<12	11 MT vs. 1 RT ^a^	19 (63.3%)	22 (73.3%)	16 (53.3%)	18 (60.0%)
	11 MT vs. 1 OT ^b^	17 (56.7%)	15 (50.0%)	9 (30.0%)	10 (33.3%)
	11 MT vs. 9.0 mL syrup	21 (70.0%)	23 (76.7%)	23 (76.7%)	28 (93.3%)
	1 RT ^a^ vs. 1 OT ^b^	12 (40.0%)	10 (33.3%)	5 (16.7%)	3 (10.0%)
	1 RT ^a^ vs. 9.0 mL syrup	16 (53.3%)	12 (40.0%)	17 (56.7%)	22 (73.3%)
	1 OT ^b^ vs. 9.0 mL syrup	18 (60.0%)	18 (60.0%)	24 (80.0%)	26 (86.7%)
12–<18	70 MT vs. 1 RT ^c^	13 (43.3%)	22 (73.3%)	15 (50.0%)	18 (60.0%)
	70 MT vs. 1 OT ^d^	11 (36.7%)	23 (76.7%)	8 (26.7%)	12 (40.0%)
	70 MT vs. 18.0 mL syrup	15 (50.0%)	18 (60.0%)	26 (86.7%)	17 (56.7%)
	1 RT ^c^ vs. 1 OT ^d^	13 (43.3%)	10 (33.3%)	1 (3.3%)	4 (13.3%)
	1 RT ^c^ vs. 18.0 mL syrup	20 (66.7%)	15 (50.0%)	25 (83.3%)	20 (66.7%)
	1 OT ^d^ vs. 18.0 mL syrup	18 (60.0%)	15 (50.0%)	28 (93.3%)	24 (80.0%)

^a^: Ø 6 mm; ^b^: 6 × 2.5 mm; ^c^: Ø 13 mm; ^d^: 14.5 × 5.7 mm; N = number of participants; MT = mini-tablet(s); OT = oblong tablet; RT = round tablet; vs. = versus.

## Data Availability

Individual participant data from this study will be available in an anonymized form upon request. Proposals should be directed to the corresponding author.
